# DeOTA-IoT: A Techniques Catalog for Designing Over-the-Air (OTA) Update Systems for IoT

**DOI:** 10.3390/s26010193

**Published:** 2025-12-27

**Authors:** Mónica M. Villegas, Mauricio Solar, Fáber D. Giraldo, Hernán Astudillo

**Affiliations:** 1 Departamento de Informática, Universidad Técnica Federico Santa María, Av. España 1680, Valparaíso 2390123, Chile; msolar@inf.utfsm.cl; 2 Faculty of Engineering, University of Quindío, Carrera 15 No. 12 Norte, Armenia 630001, Colombia; fdgiraldo@uniquindio.edu.co; 3 Instituto de Tecnología para la Innovación en Salud y Bienestar (ITiSB), Universidad Andrés Bello, Calle 1 Oriente 1180, Viña del Mar 2530959, Chile; hernan.astudillo@unab.cl

**Keywords:** Internet of Things, sensors, IoT, software architecture, quality attributes, Over-the-Air, OTA, OTA updates, OTA update systems, techniques, firmware, techniques catalog, catalog, mechanisms

## Abstract

The rapid expansion of Internet of Things (IoT) applications requires robust mechanisms to ensure the security, reliability, and maintainability of embedded software throughout its lifecycle. Over-the-Air (OTA) update systems play a central role in enabling the continuous evolution of IoT deployments. Despite their importance, OTA solutions are often designed in an ad hoc manner, supported by fragmented guidelines that lack a structured basis for selecting mechanisms and techniques aligned with the quality needs of IoT systems. This work presents a consolidated catalog for designing OTA update systems in IoT environments, developed through a review of academic and industrial literature. The catalog comprises 34 techniques organized into six mechanisms, each with representative use cases and a mapping to relevant quality attributes that make beneficial and adverse impacts explicit. The catalog was evaluated through a controlled industrial experiment involving 10 engineers, balanced between novices and experts, who designed an OTA update system for a real application scenario using either their prior knowledge and experience or the catalog. This work offers four contributions: (1) a catalog of 34 OTA techniques structured into six mechanisms; (2) clarified architectural definitions of technique and mechanism; (3) a controlled industrial experiment evaluating the catalog in a realistic setting; and (4) a quality-attribute trade-off analysis for each technique. Together, these contributions establish a coherent foundation for systematic and quality-aware OTA update system design.

## 1. Introduction

The Internet of Things (IoT) has evolved into a foundational layer of modern Cyber–Physical Systems (CPSs) [1], spanning applications ranging from mission-critical military and industrial systems to large-scale smart cities, precision agriculture, smart homes, and healthcare infrastructures [2].

Minerva et al. [3] conceptualize IoT from two complementary perspectives: (1) small-scale deployments, where identifiable “Things” embed sensing, actuation, and programmability capabilities; and (2) large-scale environments, where vast populations of interconnected devices collaborate to execute complex distributed processes. Across both scales, ensuring that devices remain continuously updated is essential to avoid malfunctioning behavior, maintain operational reliability, and mitigate evolving security threats. However, in IoT deployments, updating devices is considerably challenging due to their heterogeneity, hardware and energy constraints, and intermittent connectivity, all of which require systematic, resource-aware update mechanisms [4]. As a result, IoT ecosystems require update strategies that are not only secure and reliable but also systematic, resource-aware, and adaptable to diverse deployment conditions.

Given the centrality of update processes in IoT, a variety of approaches have been developed to distribute software and firmware revisions across deployed devices. Among these, Over-the-Air (OTA) update processes have emerged as the predominant approach for sustaining the long-term evolution of IoT systems [5]. OTA updates are architected, remotely orchestrated procedures that deliver, verify, and activate software or firmware revisions directly on embedded devices, eliminating the need for physical access. By enabling continuous and scalable maintenance, OTA updates address many of the inherent challenges of IoT deployments, supporting secure distribution, failure-tolerant installation, and coordinated management of large and diverse device fleets [5,6].

Despite their central role in IoT system dependability, OTA updates are still frequently designed in an ad hoc manner. Existing architectural guidelines remain fragmented and do not provide a clear basis for selecting solutions that align with the specific requirements and quality needs of IoT environments [5]. Although previous studies have proposed taxonomies and early attempts to organize the OTA update domain [5,7,8], the available knowledge is dispersed across heterogeneous sources and lacks integration into a coherent architectural perspective. As a result, developers often rely on isolated practices, and there is no standardized approach for incorporating security, maintainability, and other quality attributes (QAs), understood as non-functional properties that characterize how a system behaves under particular conditions [9], into OTA update systems [10]. This is particularly problematic because OTA design decisions inherently involve trade-offs among QAs such as security, availability, energy management, and flexibility. In this work, we use the term “trade-off” to describe situations where improving one quality attribute may negatively affect another, requiring architects to balance competing design concerns [9]. For instance, cryptographic verification strengthens security but increases energy consumption, while modular update strategies enhance maintainability but may introduce performance overhead [11].

To address this gap, this work consolidates the dispersed and inconsistently described body of knowledge on OTA updates into a coherent catalog of architectural techniques. Through a literature review and a structured unification process, the study identifies recurring solution elements and organizes them into a set of well-defined techniques, which are concrete design procedures that solve a specific OTA-related problem; and mechanisms, which group related techniques that collectively implement key stages of the OTA update process. To assess the catalog’s practical relevance, we conduct a controlled industrial experiment to determine whether the explicit articulation of techniques and their associated quality-attribute impacts improves the completeness, consistency, and accuracy of architectural decisions compared to unaided, experience-based design. The rigor underlying the catalog’s construction, combined with insights from the industrial study, provides an initial indication that it supports more systematic and transparent architectural decision-making for OTA-enabled update systems.

This research builds on prior work [6,8] and contributes to the body of knowledge in four ways: (1) it introduces a catalog of 34 techniques for designing OTA update systems for IoT, systematically organized into six mechanisms that structure the end-to-end update process; (2) it clarifies the notions of technique and mechanism within the OTA context, providing precise architectural definitions that have been missing from previous studies; (3) it reports an experimental validation conducted in an industrial setting, using real subjects and tasks to assess the catalog’s practical usefulness; and (4) it provides a quality-attribute trade-off analysis that evaluates each technique across key attributes such as security, scalability, performance, availability, interoperability, reliability, privacy, energy management, flexibility, and evolvability, using a 5-point Likert-style bipolar scale [12,13]. By making these trade-offs explicit, the catalog consolidates dispersed knowledge and provides architects with a structured basis for systematically and balancedly designing OTA update systems across diverse IoT contexts. Beyond these contributions, the present study significantly extends our earlier OTA taxonomy work by incorporating five additional years of evidence (2020–2025), applying a structured homologation process, introducing the first quality-attribute trade-off model for OTA techniques, and conducting the first controlled industrial experiment evaluating their impact on architectural decision-making, thereby transforming the original taxonomy into a comprehensive and empirically validated design instrument.

The remainder of this article is organized as follows. Section 2 introduces the foundational concepts, terminology, and architectural elements underlying OTA update systems. Section 3 reviews the state of the art on OTA update system design and identifies the gaps and limitations that persist in existing approaches. Section 4 outlines the research process and methods used to construct the catalog. Section 5 presents the catalog itself. Section 6 reports the experimental validation and its results. Section 7 addresses threats to validity and their mitigation. Section 8 discusses the main findings, their implications, and limitations. Finally, Section 9 summarizes the contributions and identifies directions for future research.

## 2. OTA Updates in IoT Systems

Software evolution is a fundamental architectural concern in distributed systems. Updates enable systems to correct defects, introduce new functionality, and extend capabilities over time [9]. They may target software, data, or hardware components, and can be delivered through physical interfaces or remotely across a network. In large and heterogeneous IoT environments, where devices are geographically distributed, resource-constrained, and often intermittently connected, remote delivery becomes a practical necessity for maintaining system continuity.

OTA updates are commonly defined as the remote distribution and installation of software on embedded devices by transferring update artifacts from a backend system to nodes across a network [8]. Architecturally, OTA update solutions integrate two complementary subsystems [6]. The first is the embedded software responsible for executing the update logic on each device. The second is the backend infrastructure that supports update preparation, validation, orchestration, distribution, and device coordination at scale. Together, these subsystems enable a continuous and reliable update process suitable for distributed IoT deployments.

Following the terminology introduced by Villegas and Astudillo [8], it is useful to distinguish between mechanisms and techniques when characterizing OTA update systems. A mechanism represents an architectural building block that encapsulates a specific responsibility within the update process. Mechanisms may be implemented in software, hardware, or a hybrid of both, and they define the structural and behavioral boundaries of the OTA subsystem. Examples include secure delivery, validation, installation management, scheduling, rollback control, and device orchestration. As abstractions of system functionality, mechanisms establish the interfaces through which OTA components interact and can be composed to form complete update workflows.

A technique, in contrast, denotes the concrete procedure through which a mechanism is realized. Techniques describe the operational steps used to prepare, transfer, verify, and apply updates across distributed IoT devices. They reflect engineering practices grounded in research, prevailing standards, and accumulated industry experience, and are typically adapted to the constraints of particular hardware platforms, protocols, and deployment environments. While mechanisms articulate the architectural responsibilities within an OTA system, techniques specify how these responsibilities are executed in practice.

Techniques operate at the implementation level and address operational concerns such as reliability, efficiency, scalability, and security. Each technique represents a tangible procedure with its own workflow and measurable effects on system qualities. Together, mechanisms and techniques provide a structured way to interpret the functional and operational layers of OTA update systems and to reason about the interplay between architectural responsibilities and their concrete realization.

**Figure 1:**
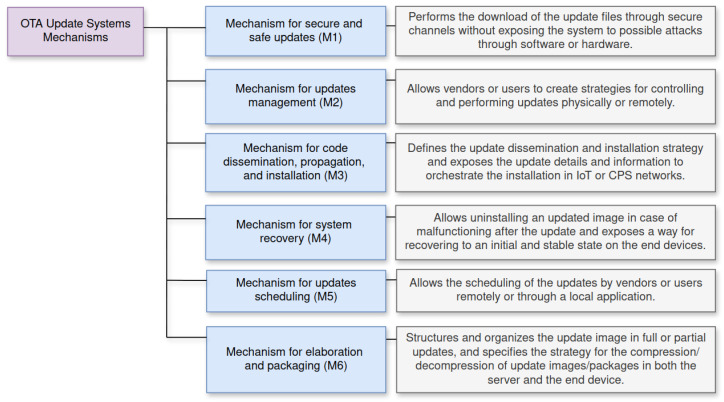
Description of the six mechanisms that set up an OTA update system for IoT, created according to the definition proposed by Villegas and Astudillo [8].

Building on this distinction, Villegas and Astudillo [8] proposed a taxonomy that organizes OTA update systems into six mechanisms, each capturing a core architectural responsibility. As summarized in Figure 1, these mechanisms cover secure update preparation, management, dissemination and installation, recovery, scheduling, and packaging, providing a clear structural view of the OTA update process.

As shown in Figure 1, the taxonomy structures OTA update systems around six architectural mechanisms that capture the main responsibilities of the update process, including secure preparation, lifecycle management, dissemination and installation, recovery, scheduling, and packaging. This classification offers a clear architectural decomposition of the OTA subsystem and clarifies how these responsibilities contribute to a coherent end-to-end update workflow.

While the taxonomy clarifies the structural organization of OTA responsibilities, understanding how these responsibilities unfold during an update requires examining their dynamic interaction. A runtime perspective exposes how mechanisms coordinate to advance the update process, how control and data flows progress through preparation, distribution, verification, installation, and recovery, and how the techniques associated with each mechanism are invoked to realize these transitions in practice.

**Figure 2:**
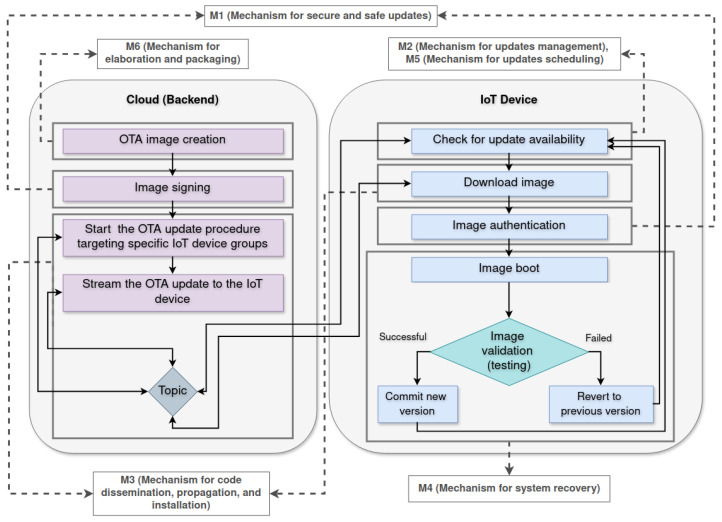
Example of an OTA Update System for IoT, in which the techniques are inside the gray rectangles, and the mechanisms they are associated with are shown as connected.

As an illustrative example, Figure 2 expands and refines the update sequence discussed by Villegas et al. [6], providing a more detailed view of how preparation, distribution, verification, installation, and recovery activities relate to one another. The figure specifies the connections between the steps of the OTA process, the techniques that operationalize each step (shown in the gray rectangles), and the mechanisms they instantiate, represented through the connecting structure.

As illustrated in Figure 2, the OTA update process is structured as a coordinated workflow that spans both the backend and the IoT device. The figure outlines the progression from image creation and signing, through distribution and verification, to installation and validation, making explicit the main control flows that govern the update lifecycle. It also shows how specific techniques instantiate the mechanisms responsible for secure delivery, update management, dissemination, and recovery. By exposing these relationships in a unified view, the diagram provides a clear architectural reference that supports reasoning about the integrity, safety, and robustness of the OTA process across heterogeneous IoT devices.

## 3. Related Work

Research on OTA update systems for IoT has advanced across several technical domains, including secure firmware delivery, dissemination efficiency, protocol and platform support, and architectural guidance. These efforts underscore the importance of OTA processes for maintaining the long-term reliability, security, and maintainability of connected devices, as highlighted by Bauwens et al. [14], Bradley and Barrera [15], and Hernández-Ramos et al. [16]. However, despite these contributions, existing work provides limited support for practitioners who require systematic, architecture-oriented methods for designing OTA update systems that must operate across heterogeneous devices, networks, and quality requirements.

A broad stream of research has concentrated on strengthening the security of OTA transmission channels. Shin and Jeon [17] propose an OTA protocol that integrates MQTT [18] with Merkle trees to enhance integrity protection during firmware distribution. Similarly, Chien and Wang [19] present an MQTT 5.0-based OTA architecture emphasizing the risks posed by insecure update pipelines to entire IoT ecosystems. In low-power wide-area contexts, Hayati [20] explores Firmware Updates Over-the-Air (FUOTA) procedures for Long Range Wide Area Network (LoRaWAN) devices [21]. Park et al. [22] further contribute a hybrid cryptographic update mechanism that combines symmetric and asymmetric primitives to safeguard firmware authenticity and confidentiality in constrained IoT devices. These studies demonstrate how secure communication channels and integrity mechanisms mitigate individual threats, but they remain confined to protocol-level or cryptographic concerns and do not offer broader architectural guidance for composing update responsibilities in a systematic manner.

A second body of work addresses efficiency and dissemination challenges in constrained environments. Malumbres et al. [23] propose hybrid unicast–broadcast dissemination strategies to reduce latency in low-end or hard-to-access devices. Mahfoudhi et al. [24] analyze update procedures for Narrowband IoT (NB-IoT) devices [25], to minimize energy and bandwidth consumption during OTA operations. Although these contributions provide valuable optimizations for specific device types and network technologies, they do not generalize into an architectural framework capable of supporting systematic decision-making across heterogeneous IoT deployments.

Complementary efforts introduce architectural abstractions and best practices. Sousa and Borin [26] present a layered OTA model emphasizing the integration of security and privacy across architectural levels. Anedda et al. [27] discuss best practices for secure and privacy-aware IoT system design, underscoring the importance of disciplined engineering approaches. Yet, these works remain high-level: they neither consolidate OTA techniques nor provide a systematic method for evaluating or selecting them according to diverse quality attributes.

Standardization efforts such as RFC 9019 [7] define a normative firmware update architecture including manifests, bootloaders, firmware consumers, and recovery elements, along with essential security and interoperability requirements. While these specifications establish foundational architectural principles, they intentionally abstain from detailing concrete techniques, implementation variability, or architectural trade-offs related to attributes such as reliability, scalability, or energy management.

Several surveys and domain analyses offer broader syntheses. Arakadakis et al. [28] examine Over-the-Air Programming (OTAP) techniques for heterogeneous and resource-constrained IoT nodes, discussing protocol-level dissemination strategies and performance constraints. Kesari et al. [29] provide an overview of OTA concepts across Cloud, edge, and hybrid environments, including Software Over-the-Air (SOTA), Firmware Over-the-Air (FOTA), rollback strategies, secure boot, and delta updates. El Jaouhari and Bouvet [5] focus specifically on secure firmware OTA methods, reviewing threat landscapes, reference architectures, and standardization initiatives. These surveys map the technological landscape but remain primarily descriptive and do not structure OTA knowledge into an architectural framework that relates techniques to quality-attribute trade-offs.

A more architecturally aligned perspective is offered by Villegas et al. [6], who analyze IoT, embedded, and CPS [1] to assess their support for OTA updates. Their study identifies platforms that incorporate OTA capabilities but does not extract reusable architectural guidance or a design-oriented catalog of techniques. Building on this, Villegas and Astudillo [8] introduced a preliminary taxonomy of OTA mechanisms and linked several techniques to each mechanism. While this taxonomy constitutes an important first step, it lacks detailed technique descriptions, does not characterize their quality-attribute implications, and does not provide systematic guidance for selecting or combining techniques during OTA system design.

Across these research strands, a clear limitation persists: none of the existing studies provide a systematic, architecture-oriented framework that organizes OTA mechanisms and techniques and articulates their associated quality-attribute trade-offs. Existing work advances isolated concerns, but it does not support architects in reasoning about how OTA techniques should be selected, combined, or adapted to meet diverse IoT requirements such as security, availability, energy management, and scalability.

## 4. Research Process and Methods

This section presents the methodological design used to identify, extract, consolidate, and refine the techniques that compose the DeOTA-IoT catalog. The research process integrates evidence from a previous study [8] with an updated and significantly broader literature review covering 2020–2025. The goal was to build a comprehensive, coherent, and technically grounded inventory of OTA update techniques for IoT systems while enabling a systematic comparison of their trade-offs across key QAs.

**Figure 3:**
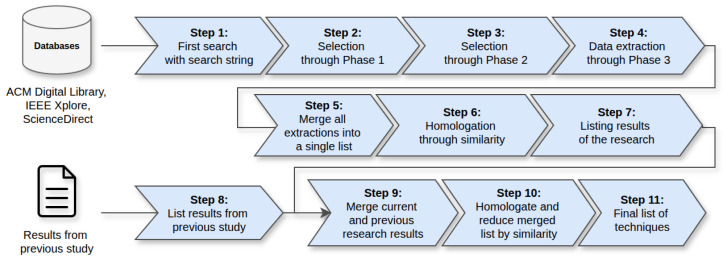
Overview of the research workflow combining new evidence from digital libraries with results from the previous study [8]. Steps 1–3 correspond to the identification and screening of relevant studies (Section 4.2.1). Step 4 extracts OTA-related techniques, while Steps 5–7 consolidate and homologate the extracted data into a unified list (Section 4.2.2). Steps 8–10 integrate results from the previous study with the new evidence and apply a second homologation pass (Section 4.2.3). Step 11 produces the final consolidated set of techniques used to construct the DeOTA-IoT catalog.

Figure 3 illustrates the overall workflow following recognized guidelines for evidence-based software engineering [30] and PRISMA recommendations [31]. It consists of eleven well-defined steps explained in three main stages: (i) literature identification and screening, (ii) extraction and homologation of techniques, and (iii) integration and consolidation of results from both the previous and current studies. Each step illustrated in Figure 3 is described in detail in the corresponding subsections of this section, following the order of the workflow.

### 4.1. Research Requirements

This subsection introduces the research questions and methodological requirements that guided the study, including the definition of the search strategy and the filtering phases used to select relevant literature.

#### 4.1.1. Research Questions

Two research questions guided the investigation:

- **RQ0:** How can OTA update techniques be systematically organized into mechanisms to support a clear and useful catalog for system designers?
- **RQ1:** How do these techniques influence critical IoT quality attributes such as security, reliability, availability, scalability, and energy management?

To answer these questions, the research adopted the following methodological actions:

1. **Evidence aggregation and technique unification (addresses RQ0):** Results from Villegas and Astudillo [8] were combined with the findings of the new review (2020–2025). All extracted techniques were merged and homologated to create a unified representation.
2. **Quality attribute impact assessment (addresses RQ1):** Each technique was evaluated by domain experts using a 5-point bipolar Likert scale [12,13], assigning impact scores based on their assessment of how each technique affects relevant quality aspects.

#### 4.1.2. Search Strategy

The literature search was designed to capture a broad range of primary studies describing OTA mechanisms, processes, and technical components for IoT systems. The search was conducted across the main digital libraries used in software engineering and IoT research, as listed in Table 1.

To retrieve studies addressing OTA updates in IoT, we used the following search string:

(OTA OR over-the-air) AND (IoT OR “internet of things”)

To select relevant material and to ensure methodological rigor, we defined inclusion/exclusion criteria for the research:

- **Exclusion Criteria:**
  – **EC1.** Texts not written in English.
  – **EC2.** Not-fully available texts.
  – **EC3.** Patents.
  – **EC4.** Not-accessible texts.
  – **EC5.** Presentations.
- **Inclusion Criteria:**
  – **IC1.** A primary study must contain software or hardware components used in the development of OTA update systems.
  – **IC2.** A primary study must have software or hardware techniques or approaches or methods for designing or implementing an OTA update process.

#### 4.1.3. Study Filtering Phases

Study selection followed a structured three-phase screening process:

- **Phase 1 (PH1):** Selection of articles in which the title and abstract were related to techniques, approaches, or methods for designing or implementing OTA update systems for IoT.
- **Phase 2 (PH2):** Selection of articles in which the introduction and proposal were related to methods, techniques, processes or approaches in the context of designing or implementing OTA update systems for IoT.
- **Phase 3 (PH3):** Extraction of the techniques used in the OTA update systems reported for IoT. The extraction method involves obtaining the technical components, either software or hardware components involved in the design or implementation process of the OTA Update system.

### 4.2. Research Process

The research process expands and enhances the previous study [8], which classified OTA techniques up to 2020 but did not perform homologation nor detail the associated trade-offs. The present study incorporates five additional years of research and applies a structured, multi-step process to refine a unified and comprehensive catalog.

#### 4.2.1. Literature Identification and Screening

*Step 1* executed the multi-library search. *Steps 2 and 3* applied PH1 and PH2 filters, ensuring that only studies directly relevant to OTA updates in IoT were retained.

**Figure 4:**
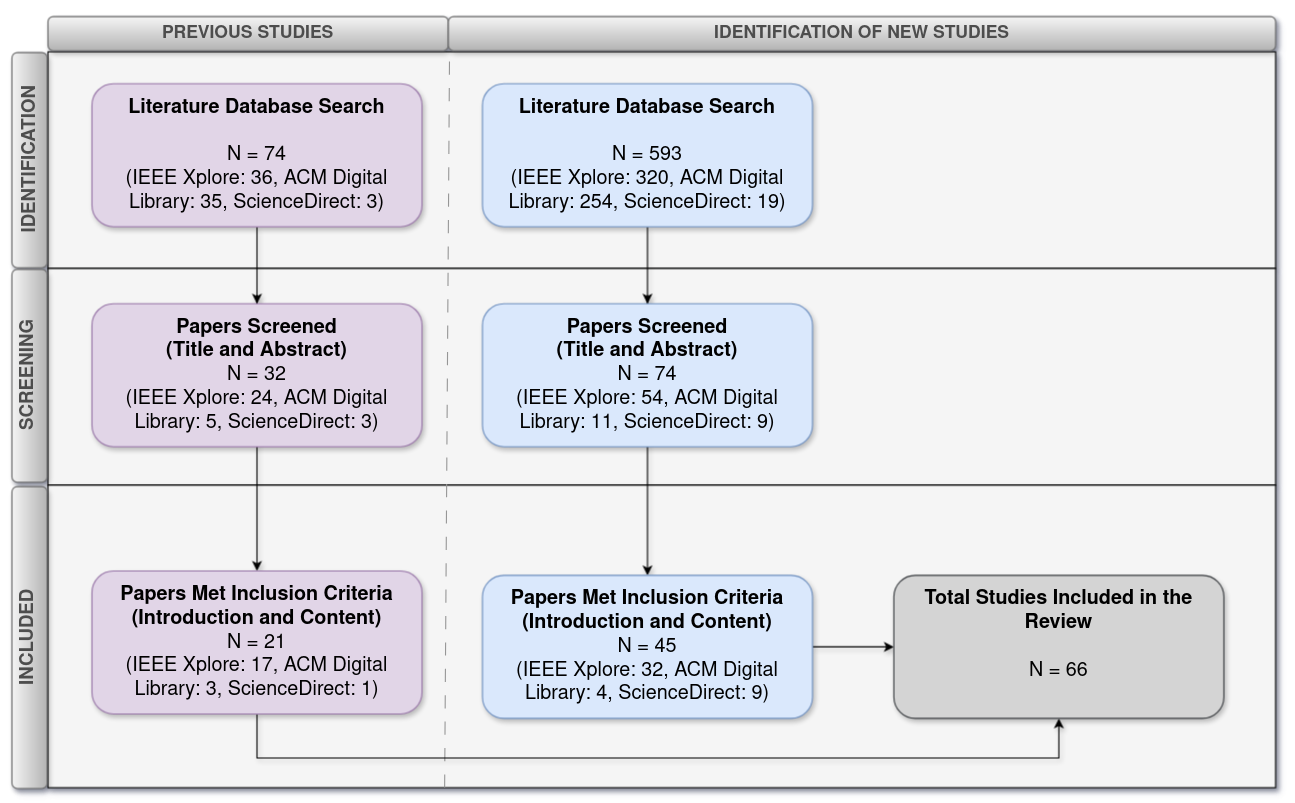
Study identification, screening, and inclusion across the different digital libraries.

Figure 4 illustrates the number of studies identified, screened, and included after applying the filtering phases.

#### 4.2.2. Technique Extraction and Initial Homologation

*Step 4* extracted OTA-related techniques based on PH3. *Step 5* merged all extracted data into a unified dataset. *Step 6* conducted the first homologation pass, consolidating techniques exhibiting functional equivalence, similar operational goals, and differences limited to naming or implementation context. *Step 7* produced a structured list of techniques representing the updated evidence base for 2020–2025.

#### 4.2.3. Integration with Previous Evidence and Final Consolidation

*Step 8* reorganized techniques identified in the previous study [8]. *Step 9* integrated prior and new datasets. *Step 10* applied a second homologation pass to eliminate deep redundancies and ensure conceptual coherence across both periods. *Step 11* produced the final set of 34 techniques after a rigorous multi-stage consolidation process.

To transform this heterogeneous collection into a coherent and architecturally meaningful catalog, we applied two consecutive homologation passes. We first consolidated techniques exhibiting functional equivalence across the reviewed sources [5,28,32,33,34,35,36,37,38,39,40,41,42,43,44,45,46,47,48,49,50,51,52,53,54,55,56,57,58,59,60,61,62,63,64]. We then refined the resulting set by grouping techniques according to their architectural role and intended objective within the OTA update process. Redundant, overlapping, or overly specialized entries were merged into unified, semantically consistent techniques that preserved their essential functional intent.

The result of this refinement is a distilled set of 34 techniques, each representing a clear, well-defined, and architecturally significant approach within the DeOTA-IoT catalog.

### 4.3. Quality Attributes Identification and Selection

To evaluate the architectural impact of each technique, we adopted the set of IoT quality attributes proposed by Khezemi et al. [65], which synthesizes 103 primary studies and provides a domain-independent perspective on IoT software quality. These attributes include security, reliability, availability, scalability, performance, interoperability, privacy, energy management, evolvability, and flexibility.

This selection aligns with the definition of QAs as system properties measurable during architectural evaluation [9] and complements ISO/IEC 25010 quality characteristics [66].

### 4.4. Trade-Off Identification and Impact Representation

This subsection describes the process for identifying and characterizing the architectural trade-offs associated with each OTA technique. It explains how quality-attribute impacts were elicited, validated, and consolidated into a comparative model supporting RQ1.

#### 4.4.1. Assessment Method

Trade-offs were evaluated using a 5-point bipolar scale [12,13], grounded in the formal theory of bipolar measurement. This type of scale enforces symmetry between negative and positive categories, with zero representing a meaningful and cognitively stable midpoint. As shown by Demin et al. [12], equidistant categories and a coherent numerical structure are essential for capturing direction-sensitive judgments without distorting their interpretation.

The scale used in this study ranged from −2 (degradation) to +2 (improvement), with 0 denoting no discernible impact. Table 2 summarizes the interpretation of each value for QA impacts.

This configuration is particularly well-suited to OTA techniques, whose architectural effects are rarely one-sided: a technique can strengthen one quality attribute while simultaneously stressing another. The bipolar model provides the precision and balance needed to expose these competing forces and reason cleanly about the resulting trade-offs.

#### 4.4.2. Expert-Based Evaluation

To ensure the validity and practical relevance of the impact assignments, the assessment relied on four senior professionals with 13–17 years of experience in Software Architecture, IoT, Information Security, and IT Management. Their experience in designing and operating distributed systems enabled them to interpret architectural implications beyond those explicitly reported in primary studies. Table 3 summarizes the experts involved in the quality-attribute trade-off assessment.

#### 4.4.3. Evaluation Procedure

The evaluation followed a streamlined yet rigorous three-step procedure:

1. **Independent scoring**: Each expert rated all technique–QA pairs independently, ensuring unbiased reasoning and capturing variation in practitioner perspectives.
2. **Consensus session**: A focused 60-min session was held to review divergences, clarify architectural assumptions, and converge on stable, shared interpretations of the impacts.
3. **Stability verification**: A final cross-check ensured consistency across mechanisms, eliminated asymmetries in the use of the scale, and confirmed adherence to the semantics of a strictly bipolar model.

The three steps ensured impact assessments that are internally consistent, analytically balanced, and grounded in expert architectural judgment.

#### 4.4.4. Resulting Trade-Off Model

The consolidated ratings form a comparative trade-off model that makes explicit where each OTA technique provides value, where it introduces tension, and how it affects the overall quality-attribute landscape. This model directly supports RQ1 by enabling systematic comparison across techniques and their architectural trade-offs.

Through structured evidence aggregation, multi-stage homologation, and expert-driven refinement, the methodological pipeline consolidates a heterogeneous body of OTA-related practices into a coherent and architecturally meaningful set of techniques. Rather than being a mere byproduct of synthesis, this set represents a distilled view of recurring responsibilities, operational patterns, and architectural approaches. As a result, it provides a stable, reproducible, and conceptually grounded basis for characterizing the solution space of OTA update systems in IoT.

## 5. The DeOTA-IoT Techniques Catalog

The catalog presented in this section is the consolidated output of the methodological process described previously. By integrating evidence from the literature, harmonizing terminology, and validating the resulting abstractions with experts, the study distilled a broad set of OTA practices into a structured and architecturally meaningful collection of techniques. The catalog follows the six mechanisms that define the core responsibilities of OTA update systems for IoT, ensuring a direct alignment between architectural intent and operational realization.

The catalog is designed to serve both as a practical reference and as an analytical instrument. It supports engineers in selecting and combining OTA procedures, and it enables systematic reasoning about how these procedures affect system-level qualities in heterogeneous IoT deployments.

### 5.1. Techniques Characterization

Each technique is described using a uniform specification template that captures: (1) a concise title; (2) a technical description that clarifies its operational logic and architectural role; (3) representative use cases; (4) a workflow representation; and (5) an impact assessment over the selected QAs.

The techniques specified in the catalog and grouped into the six mechanisms are the following:

1. **Mechanism for secure and safe updates (M1):**
  - **T.1.1 Firmware Integrity, Authenticity, and Installation Verification:** Use cryptographic hashes, such as SHA-256 or CRC [67], and digital signatures, such as ECDSA or RSA [67], to verify firmware integrity during transfer. Also, verification should be performed within trusted environments, and attestation mechanisms should be applied to confirm that the installation has been completed successfully. Secure boot mechanisms, such as ARM TrustZone, Secure Boot System Firmware Update (SBSFU) [68], or similar solutions, are used to ensure the authenticity of firmware before it is executed.
  - **T1.2 Secure Payload Encryption and End-to-End Communication:** Firmware payloads should be encrypted with symmetric or asymmetric cryptographic algorithms, such as AES or RSA [67], and packaged in secure formats, for example, CBOR Object Signing and Encryption (COSE) [69], JSON Object Signing and Encryption (JOSE) [70], or JSON Web Signature (JWS) [71]. OTA transmissions should be protected using protocols such as Transport Layer Security (TLS), Datagram Transport Layer Security (DTLS), or Object Security for Constrained RESTful Environments (OSCORE) [72]. The use of short-lived credentials helps to maintain confidentiality, authenticity, and resilience during communication between the Original Equipment Manufacturer (OEM) and the device.
  - **T1.3 Trusted Execution and Runtime Protection:** Safeguard the integrity and confidentiality of the OTA update process during installation and execution by isolating sensitive operations within secure, trusted, or hardened execution environments [73,74,75]. Secure environments include Trusted Execution Environments (TEEs), hypervisors, or hardened operating system contexts, which specifically protect cryptographic keys, update logic, and system state. By isolating these components, sensitive processes and data are shielded from compromise throughout both installation and execution phases, particularly important for devices exposed to physical access or hostile runtime conditions.
  - **T1.4 Key, Credential, and Identity Management:** Generate, provision, and manage cryptographic keys and digital certificates to facilitate secure device-server authentication [74,75]. The system must maintain unique device identities, and robust authentication credentials, such as multi-factor authentication or SIM-based methods, should be pre-installed and configured. Additionally, the System must enforce role-based access control.
  - **T1.5 Decentralized and Blockchain-Based Verification:** Use blockchain, smart contracts, fog nodes, or similar decentralized methods [76,77,78,79] to verify firmware authenticity, track delivery, enforce constraints, and allow devices to perform transaction-based validation queries before installation.
  - **T1.6 Compatibility and Threat Prevention:** Perform compatibility checks via binary analysis or simulation/digital twins before deployment [80]. Detect and prevent threats using gateway-based anomaly detection, device fingerprinting, and network isolation to block unauthorized updates.
  - **T1.7 Protocol, Debug Interface, and Request Security:** Prevent unauthorized update requests, protocol-level attacks, and exploitation of debug interfaces by securing communication protocols and restricting low-level system access using timestamp validation, integrating link-layer security (e.g., AUTOSAR Secure Onboard Communication (SecOC) [81]), securing debug interfaces (e.g., JTAG [82], SWD [83]), and authenticating update requests using tokens, challenge–response, or Multi-Factor Authentication (MFA) [84]. This technique addresses threats such as replay attacks, unauthorized firmware injection, and misuse of debug ports, ensuring that OTA update requests follow authenticated and validated communication paths.
  - **T1.8 Installation and Runtime Security Assurance:** Maintain long-term firmware integrity and system trust by enforcing secure installation conditions and continuous runtime verification using certificates, Root of Trust, Physically Unclonable Functions (PUFs), TEE storage, and short-lived tokens to secure installation processes and maintain firmware integrity during runtime [85]. This technique establishes a persistent root of trust that ensures only authenticated and validated firmware remains active across reboots and update cycles, supporting sustained system reliability and resistance to post-installation tampering.
2. **Mechanism for updates management (M2):**
  - **T2.1 OTA Orchestration and Management Interfaces:** Use centralized or distributed OTA platforms (e.g., hawkBit [86], ZT-OTA [64]), web/mobile dashboards, and lifecycle APIs (RESTful CRUD, polling, event triggers) [87] to manage firmware updates, deployment, and logging.
  - **T2.2 Role-Based Access and Permissions:** Define update actors (e.g., OEM, integrator, uploader, user) and assign role-based permissions to control who can initiate, approve, or manage firmware updates.
  - **T2.3 Workflow Coordination and Traceability:** Manage the update process through structured workflows (submission, review, authorization, deployment) with unique session identifiers for tracking and conflict prevention.
  - **T2.4 Device Classification and Delivery Mode Selection:** Categorize devices by capability (secure-capable vs. constrained) and choose appropriate delivery methods (WiFi direct, BLE to WiFi, BLE-only) to optimize update efficiency [7].
  - **T2.5 User Awareness and Consent Management:** Notify users before updates, allow them to approve or postpone installations, and provide clear summaries of changes before and after the update.
3. **Mechanism for code dissemination, propagation, and installation (M3):**
  - **T3.1 OTA Protocols, Delivery Models, and Channel Selection:** Disseminate updates using MQTT, CoAP, HTTP(S), BLE mesh, LoRaWAN multicast, satellite, wired, or hybrid push–pull approaches [7,88]. Select centralized, gateway, peer-to-peer, or decentralized (e.g., IPFS [89]) models based on scalability, reach, and device capabilities.
  - **T3.2 Manifest-Driven and Policy-Based Update Control:** Use dedicated channels for manifest distribution, including metadata, signatures, version rules, rollback constraints, and attestation data. Support deferred or event-triggered installation and coordinate updates with middleware or decentralized logic enforcing policies across devices.
  - **T3.3 Reliable Transfer and Execution Resilience:** Ensure that OTA updates are delivered and applied correctly despite network instability, device restarts, or power interruptions. This technique incorporates operations such as acknowledgments, buffering, retries, resumable transfers, and execution checkpoints to prevent partial or corrupted updates. It is especially relevant in large-scale or unreliable network environments, where resilience is required to preserve system availability and prevent inconsistent device states.
  - **T3.4 Pre-Deployment Validation and Optimization:** Perform compatibility checks, sandbox testing, and module validation before deployment. Enhance targeting and performance using TinyML-based deployment [90], model-specific packaging, anomaly detection, and predictive optimization.
  - **T3.5 Lightweight Incremental Update Application:** Reduce update size, transmission overhead, and installation time by applying firmware or software updates incrementally rather than replacing full images. This technique updates only the modified portions of the system through insert, modify, delete, or copy operations in memory, making it particularly suitable for bandwidth-constrained, energy-limited, or intermittently connected IoT devices. Lightweight incremental updates improve availability and energy efficiency by minimizing update duration and reducing disruption during the update process.
  - **T3.6 Secure Transfer, Authentication, and Installation Readiness:** Protect the OTA dissemination and installation process against unauthorized access, replay attacks, and unsafe installation conditions. Use methods such as challenge–response, One-Time Password (OTP), bound tokens, certificates, mutual authentication, or JSON Web Token (JWT) [91]. Monitor system readiness and manage update state transitions using state machines. Prevent rollback by employing write-once memory. These measures directly reduce risks such as unauthorized modification, downgrading critical software, and installing updates on unprepared devices. By combining authentication methods, update state management, and rollback prevention, the likelihood of malicious updates, software version inconsistencies, and system instability during dissemination is reduced.
4. **Mechanism for system recovery (M4):**
  - **T4.1 Partition-Based Rollback and Recovery:** Use A/B partitioning, checkpointing, Non-Volatile Memory (NVM) [92], and bootloader switching to enable firmware rollback and system recovery after failed updates.
  - **T4.2 Telemetry-Driven Recovery Triggers:** Monitor update outcomes via telemetry and version checks, initiating rollback if errors or inconsistencies are detected.
  - **T4.3 Role-Based Recovery Initiation:** Allow authorized roles, such as firmware uploader or manufacturer, to initiate recovery using dashboards or management tools.
  - **T4.4 Energy-Aware and Selective Retransmission:** Implement recovery strategies that minimize energy consumption by using selective retransmission for failed update segments.
  - **T4.5 Pre-Commit Testing and Post-Recovery Validation:** Conduct update code testing before committing changes and verify system stability after recovery to ensure proper functionality.
5. **Mechanism for updates scheduling (M5):**
  - **T5.1 Context-Aware Update Scheduling:** Schedule updates based on battery thresholds, GPS or network time synchronization, retry windows, and deferred execution slots.
  - **T5.2 Event and Period-Based Update Triggers:** Initiate updates via specific events, such as repository merges, or through periodic checks of the gateway version.
  - **T5.3 Multi-Period Update Arrangement:** Organize updates into multiple periods with the ability to pause or resume specific update modules.
  - **T5.4 Role-Based Scheduling Authorization:** Enforce scheduling permissions through dashboard role assignments and require device registration/authentication before scheduling.
  - **T5.5 Policy-Driven Scheduling Enforcement:** Enforce update scheduling and execution policies based on user role, device status, or operational safety constraints.
6. **Mechanism for elaboration and packaging (M6):**
  - **T6.1 Standardized Packaging and Metadata:** Use standard update formats (SWUpdate [93], LwM2M [94]) with structured metadata (JSON [95], FlatBuffers [96]) and delta compression for efficient update creation.
  - **T6.2 Modular Firmware Update Strategies:** Implement runtime linking, in-place updates, and dependency resolution to enable modular and flexible firmware updates.
  - **T6.3 ML Model Packaging for OTA:** Package OTA-delivered machine learning models with embedded metadata to ensure compatibility and traceability.
  - **T6.4 Storage Optimization via Compression and Caching:** Use compression and flash-based caching to minimize storage usage during updates.
  - **T6.5 Secure and Optimized Payload Formats:** Apply compression, delta updates, or custom payload formats with integrated versioning and encryption for secure, efficient delivery.

Each technique in the catalog is defined as an architectural decision unit, rather than as a single implementation step. In particular, techniques in the security and dissemination mechanisms may encompass multiple related practices because they address a common architectural purpose and are typically selected and evaluated together during OTA system design. For this reason, technique descriptions explicitly state not only how a solution is realized, but also why it is applied and in which contexts it is most relevant. This framing is intended to support architectural reasoning, comparison, and justification of design choices without committing to low-level implementation details at early design stages.

The implications of these characterizations are made explicit through the trade-off representation described in Section 5.3.

### 5.2. Quality Assessment Specification

As established in Section 4.3, the catalog uses ten quality attributes to represent how each technique affects these qualities within the architecture of an OTA update system. These attributes reflect system-level concerns that shape the design, coordination, and execution of OTA update procedures and provide a common vocabulary for characterizing the techniques across mechanisms.

Table 4 presents the selected attributes together with their definitions. The definitions emphasize architecturally observable properties to support consistent expert assessment and comparative reasoning across techniques. This table consolidates the terminology used throughout the catalog and clarifies the scope of each attribute before it is applied to describe the quality implications associated with the techniques.

These attributes define the quality dimensions adopted in the catalog and complete the specification of the criteria used to characterize the techniques. With these definitions in place, the foundation is set for examining how each technique influences the quality profile of an OTA update system.

The quality assessment performed in this study is intentionally grounded in software architecture quality attributes, which provide a well-established basis for reasoning about architectural trade-offs. Accordingly, the assessment focuses on how OTA techniques positively or negatively influence attributes such as security, scalability, performance, availability, interoperability, reliability, privacy, energy management, flexibility, and evolvability.

Economic cost and implementation time are not treated as quality attributes within this specification. While these factors are undeniably important in engineering practice, they are highly context-dependent and vary according to organizational structures, existing infrastructure, development processes, tooling ecosystems, and team expertise. As a result, they cannot be consistently or objectively characterized at the architectural technique level across heterogeneous IoT contexts. For these reasons, economic and implementation cost modeling is considered outside the scope of the present quality-oriented assessment and is identified as an important direction for future work, complementing the architectural insights provided by the DeOTA-IoT catalog.

### 5.3. Characterization and Representation of Architectural Trade-Offs

The impact assignments derived from the expert evaluation are integrated into the catalog to make the architectural consequences of each technique explicit. These values indicate how every technique influences each quality attribute and form a comparable impact profile across mechanisms.

Table 5 compiles these ratings in a unified structure. Each cell reflects the expert-validated effect of a technique on a specific attribute, enabling direct comparison and exposing the contrasting quality tendencies that characterize OTA update solutions. By presenting the impacts in this consolidated form, the catalog reveals the trade-offs inherent to OTA design and provides a clear basis for selecting and combining techniques according to system-level objectives.

#### Role of Trade-Off Characterization in the Design Procedure

The trade-off characterization presented in this subsection operationalizes the DeOTA-IoT catalog as a decision-support instrument rather than a descriptive listing of techniques. During OTA system design, architects typically select candidate techniques within each mechanism based on functional requirements and deployment constraints. The trade-off profiles provided in Table 5 enable designers to explicitly evaluate how each candidate technique strengthens or stresses relevant quality attributes, such as security, availability, energy management, or evolvability.

In the overall procedure, trade-off characterization serves three complementary purposes. First, it supports intra-mechanism comparison, allowing architects to contrast alternative techniques that fulfill the same architectural responsibility but exhibit different quality impacts. Second, it enables cross-mechanism reasoning, making visible how the combined selection of techniques may introduce reinforcing or conflicting quality effects across the OTA workflow. Third, it provides a transparent rationale for architectural decisions, supporting justification, communication, and later reassessment as system requirements evolve.

This explicit representation of trade-offs was directly leveraged in the experimental validation, where participants in the experimental group used the catalog not only to identify relevant techniques but also to reason about their suitability given the scenario’s quality requirements. In this sense, the trade-off model is an integral component of the catalog-driven design process, linking evidence-based technique selection with systematic, quality-aware architectural reasoning.

## 6. Experimental Validation

To assess the practical value of the catalog as a design support instrument for OTA update systems, we conducted a controlled experiment in an industrial setting. The study examined whether the catalog enables practitioners to produce OTA architectures that are more complete, accurate, and internally consistent than those derived solely from professional expertise.

Industrial experiments commonly operate with reduced groups due to availability and operational constraints in companies [97]. In such settings, small sample sizes are standard, and robust effect-size estimators provide stable insights despite small *n* [98]. For this reason, although the experiment involved only ten participants, the analysis does not rely on statistical significance testing, whose power would be insufficient for the sample size. Instead, the evaluation centers on performance metrics and nonparametric effect-size estimators such as the Vargha–Delaney $A_{12}$ [99] and Cliff’s δ [100]. These estimators provide distribution-free assessments of practical differences between conditions and are well-suited to small-sample experimental designs. Their use allows the study to quantify the magnitude and direction of the observed effects and to assess whether the empirical patterns reflect practically meaningful differences between the evaluated conditions.

### 6.1. Experimental Design

The experimental design establishes the controlled setting for evaluating the catalog’s contribution to OTA architectural decision-making. It defines the hypotheses, participant groups, tasks, materials, and evaluation criteria, following established empirical software engineering guidelines [97,101].

#### 6.1.1. Objectives and Hypotheses

The objective of the experiment was to determine whether the catalog measurably improves architectural decision-making in OTA update systems.

The hypotheses guiding the experiment are:

**H0:**  *The use of the catalog does not have a significant impact on the quality or efficiency of OTA system design.*

**H1:**  *The use of the catalog significantly enhances the quality and efficiency of OTA system design, leading to more systematic decisions that align with key quality attributes such as security, maintainability, and interoperability.*

#### 6.1.2. Context and Participants

The experiment was conducted at Coffee Electronics Pte. Ltd., a multinational company specializing in embedded and IoT engineering, at its main operational center in Colombia. All members of the technology division were invited to participate, and the ten professionals who volunteered met the inclusion criteria of prior experience in distributed systems or IoT development. All participants met the inclusion criteria of having prior professional experience with distributed systems or IoT development, and no participants were excluded after enrollment.

The participant pool reflects the typical composition of an industrial IoT engineering team. Their backgrounds spanned software development, firmware development, embedded systems, and cloud–IoT integration, with experience levels ranging from early-career practitioners to senior engineers with more than a decade of industry experience. Table 6 summarizes the participants, their experience, and the group labels used later in the experimental setup.

#### 6.1.3. Experimental Setup

Participants were allocated to two experimental conditions using a stratified randomization procedure [101] based on seniority. This approach balanced junior and senior practitioners across groups, reducing the likelihood that experience-related differences would influence the outcomes.

The two groups were defined as follows:

- **Control group (G_1_):** Completed the OTA design task without access to the catalog and relied exclusively on their prior knowledge and professional experience.
- **Experimental group (G_2_):** Completed the same task with access to the catalog and received a short orientation on its structure and intended use.

Both groups addressed the same OTA design scenario, which involved heterogeneous devices, intermittent connectivity, confidentiality and integrity constraints, authenticity verification, and rollback protection. The task required participants to analyze this scenario and identify the architectural techniques they considered appropriate for addressing it.

The experimental session lasted 110 min and followed a structured sequence of activities to ensure consistent conditions across groups. This duration was deliberately selected to support reflective architectural reasoning while remaining feasible within an industrial setting. A session length of this magnitude is consistent with established practices in controlled industrial experiments in software engineering [102,103,104], where durations of 90–120 min are commonly used to balance task complexity, cognitive load, and participant fatigue. By regulating the flow of information, standardizing materials, and allocating dedicated time for independent reasoning, the session design ensured that the collected data reflected deliberate architectural judgment rather than procedural artifacts.

During the session, all participants worked individually under controlled conditions, using identical materials and tools. The session was monitored to ensure procedural consistency, mitigate bias, and maintain data reliability throughout all phases of the experiment.

Table 7 summarizes the procedure, including the content and duration of each phase of the session.

All materials used in the experiment, including the scenario description, templates, and supporting documentation, are publicly available at Zenodo (https://doi.org/10.5281/zenodo.17404292, 23 November 2025), ensuring full reproducibility.

#### 6.1.4. Scenario: LaboTech Remote Lab System

This subsection presents the scenario used as the foundation for the experimental validation. It defines the system, its operational environment, and the conditions that motivate its update needs, providing the realistic context against which architectural decisions and OTA technique selections were evaluated.

**(a)**  **System Description**

The system requiring an OTA update mechanism consists of multiple remote laboratory stations deployed across teaching facilities. Each station operates as a host PC, accessible remotely by students and instructors, and is responsible for controlling a heterogeneous set of laboratory instruments. Typical equipment connected to each station includes digital oscilloscopes, programmable power supplies, arbitrary waveform generators, cameras, and auxiliary electronic boards, interfaced through USB, LAN, and UART connections.

Each laboratory station runs a full operating system and integrates multiple software layers, including operating system packages, device drivers, manufacturer-provided SDKs, firmware update utilities for connected instruments, and experiment-control scripts developed in languages such as Python and Bash. As a result, the OTA update system must support updates to operating system components, application software, drivers, scripts, and firmware for external instruments.

The number of stations and the specific mix of instruments may vary across laboratories, reflecting realistic heterogeneity in hardware revisions, firmware versions, and connectivity constraints. This variability was intentionally preserved in the scenario to evaluate architectural decision-making in OTA under conditions representative of real industrial and academic remote laboratory environments.

The scenario represents a class of remote laboratory deployments rather than a single fixed installation.

**(b)**  **Users and Usage**

Students, professors, and technical assistants reserve time slots between 08:00 h and 22:00 h. The stations usually remain inactive during the night, although during exam periods, some classes extend late into the evening. During the sessions, data and video are transmitted in real time, and the results are stored in a shared network folder.

In the experiment, participants used this usage context to design an OTA update solution at the architectural level by selecting and justifying mechanisms and techniques, rather than implementing or configuring a concrete system.

**(c)**  **System Requirements**

The following are the main properties that the system must exhibit:

- **Performance:** The OTA system must maintain optimal performance by executing update-related activities without degrading the normal operation of the laboratory stations, particularly during active user sessions. This includes avoiding noticeable delays, excessive CPU or memory consumption, network congestion, or interference with real-time data acquisition and video streaming. Update mechanisms should therefore be designed to operate in the background, leverage incremental or staged updates, and respect scheduling constraints to ensure that ongoing experiments and remote interactions remain responsive and uninterrupted.
- **Availability:** The OTA update infrastructure must ensure that update packages, repositories, and update servers remain accessible when required during the update process. This includes maintaining the availability of distribution endpoints to prevent update failures caused by server downtime, transient connectivity issues, or incomplete package retrieval. The OTA process should support retry, resume, or fallback mechanisms to tolerate the temporary unavailability of update services without compromising the consistency of deployed devices.
- **Interoperability:** The OTA system must support updates across heterogeneous platforms, operating systems, applications, and subsystems using standardized or widely adopted protocols and interfaces. This includes accommodating diverse hardware architectures, communication stacks, and software environments while minimizing the need for platform-specific customization, thereby enabling consistent update management across all laboratory stations.
- **Security:** The OTA system must ensure that updates are applied using secure mechanisms that protect against unauthorized access, tampering, and malicious attacks affecting devices, software components, and communication channels. This includes enforcing authentication and authorization of update sources, verifying the integrity and authenticity of update artifacts, and safeguarding update processes against replay, rollback, or man-in-the-middle attacks to preserve system trustworthiness.

**(d)**  **Factors Driving Frequent Updates**

- Changes in the semester curriculum (new experiment scripts, new driver versions, and the addition of instrument models).
- Application of security patches for the operating system, runtime environments, and remote access components.
- Firmware updates for instruments are provided by manufacturers (installed using the host utilities).
- The university requires updates to security certificates and the VPN client for remote access.

**(e)**  **Updatable Artifacts**

- **Host operating system packages**: These include the Linux kernel or cumulative operating system updates.
- **Runtime environments and libraries**: Python 3.14 environments, software packages, Java libraries, and associated scripts.
- **Experimental software**: Applications, code repositories, configuration files, and experiment templates.
- **Instrument firmware**: Updated using manufacturer tools over USB or LAN; sometimes requires exclusive access and device reboot.
- **Drivers**: USB adapters, camera capture drivers, and kernel modules.
- **Configurations and policies**: Device whitelists, instrument assignment settings, reservation schedules, logging levels, and VPN profiles.
- **Licenses**: License files and firmware for USB keys (dongles) used with specific SDKs.

**(f)**  **Environment Constraints**

- **Topology**: USB hubs connected in chains; occasionally, faulty cables; users may turn instruments off.
- **Heterogeneity**: Multiple instrument revisions across rooms; the exact model may have different baseline firmware versions.
- **Network**: Use of campus restrictions; some laboratories are in segmented VLANs; limited nighttime bandwidth; some buildings require a proxy.
- **Storage**: Limited free space due to raw data dumps.
- **Remote access**: Remote access tools must remain available; students should not see installer pop-up windows.

**(g)**  **Operational Rules**

- **No interruptions during reservations**: Updates can only be performed during maintenance windows or when the station is inactive.
- **Safety**: During any instrument firmware update, outputs must remain de-energized (power-supply output off, loads disabled).
- **Atomicity**: Experimental scripts and their associated runtime versions must be updated together.
- **Compatibility control**: Firmware/driver combinations for instruments are subject to a model- and revision-specific compatibility matrix.
- **Human factors**: Laboratory staff may postpone a station update by up to 24 h with short notice (due to exams or demonstrations).

The scenario intentionally abstracts away exact device counts and hardware models to focus the experimental task on architectural reasoning about OTA mechanisms and techniques rather than on platform-specific implementation details.

#### 6.1.5. Ground Truth Construction and Comparison Model

To ensure methodological rigor and analytical consistency, a Ground Truth (GT) was established as the canonical reference model for this experiment. The GT encapsulates the set of techniques that represent an expert-level solution for the OTA update scenario in IoT systems.

The complete universe of techniques available for consideration is denoted as *U*. This universe comprises the full catalog of OTA-related architectural techniques, which collectively span reliability, confidentiality, integrity, maintainability, and scalability concerns:

(1) U={T1.1,T1.2,…,T1.8,T2.1,…,T6.5}

To construct the GT, the expert panel followed a structured, multi-stage consensus protocol designed to ensure rigor, transparency, and stability in the resulting configuration. Each expert first reviewed all techniques in the universe *U* independently, assessing their necessity, relevance, and contribution to the quality attributes required by the scenario. These individual assessments were then compared to identify divergences and areas of disagreement. Through facilitated discussion, the panel examined the assumptions behind each position, considered scenario-specific constraints, and trade-offs. Using evidence-based argumentation and iterative refinement, the experts resolved inconsistencies and converged on a minimal but sufficient set of techniques deemed indispensable for achieving a secure and dependable OTA update system.

This consensus protocol was operationalized through a sequence of workshops involving the experts listed in Table 3. During these sessions, each candidate technique in *U* was analyzed in terms of its contextual relevance, architectural implications, and contribution to the target quality attributes. The process continued until unanimous agreement was reached on the final configuration representing the canonical solution:

(2) $$\begin{array}{cc} GT=\{ & T1.1,T1.2,T1.3,T1.4,T1.6,T1.7,T1.8,T2.1,T2.4,T3.1,T3.3,T3.4,\\ \; & T3.5,T3.6,T4.1,T4.3,T4.5,T5.5,T6.2,T6.3,T6.4\;\} \end{array}$$

Since the control group did not have access to the catalog during the experimental task, a subsequent mapping phase was supported by the same expert panel to ensure methodological equivalence. In this process, the experts analyzed the control group’s responses, interpreting each expressed concept and associating it with the technique from the catalog that was most semantically aligned. This mapping ensured that both groups could be represented within the same formal domain *U*, thus eliminating ambiguity and enabling valid comparative analysis.

Each participant *p* generated a subset of techniques reflecting the choices made during the design session. We denote this set as $S_{p}$, where $S_{p}\subseteq U$, indicating that $S_{p}$ contains exactly the techniques from the universe *U* that were selected by participant *p*. Because each participant reasons independently, the resulting subsets differ across participants even though the design space remains shared.

The relationship between each participant’s subset $S_{p}$ and the GT provides the basis for quantifying how closely individual design decisions align with expert architectural rationale. To operationalize this comparison, we characterize participant selections using the standard four categories of set-theoretic alignment: true positives, false positives, false negatives, and true negatives. These categories are defined formally as follows:

(3) $$\begin{array}{cc} TP_{p} & =|S_{p}\cap GT|, \end{array}$$

(4) $$\begin{array}{cc} FP_{p} & =|S_{p}\setminus GT|, \end{array}$$

(5) $$\begin{array}{cc} FN_{p} & =|GT\setminus S_{p}|, \end{array}$$

(6) $$\begin{array}{cc} TN_{p} & =|U\setminus (GT\cup S_{p})| \end{array}$$

Formally, true positives (TP) denotes techniques correctly aligned with the GT, false positives (FP) those incorrectly introduced, false negatives (FN) the relevant techniques omitted, and true negatives (TN) the non-essential ones correctly excluded. The variables TP, FP, FN, and TN form the analytical basis for the performance metrics discussed in the next section.

#### 6.1.6. Performance Metrics

To assess the extent to which the proposed catalog influences the quality of architectural decisions in OTA system design, this study employs four performance metrics used in prior empirical evaluations of architectural decision-making: Precision, Recall, F1, and Accuracy [102,103,104]. These metrics provide complementary views on participants’ ability to select techniques that align with the predefined GT.

Although the experiment is exploratory and does not involve statistical hypothesis testing due to its small sample size, these quantitative indicators offer a robust basis for examining whether the evidence supports or contradicts the defined hypotheses. Accordingly, they are appropriate for evaluating the practical influence of the catalog in this controlled setting. Table 8 summarizes their definitions and the notation used throughout the analysis.

At the analysis stage, the values of Precision, Recall, F1, and Accuracy will be summarized using descriptive statistics (mean, median, and standard deviation) and compared across groups using non-parametric effect-size measures. In particular, the Vargha–Delaney $A_{12}$ statistic [99] and Cliff’s δ [100] will be used to quantify the magnitude of the differences between G_1_ and G_2_. These measures are appropriate for small-sample, non-normal datasets and provide a robust indication of practical differences between groups without requiring formal hypothesis testing [98].

### 6.2. Experimental Results

This section presents an empirical analysis of how participants approached the OTA design task and how their decisions converged toward the established ground truth. The experimental results are based on the techniques selected by each participant for the given scenario, together with the written justifications they provided to explain their architectural decisions and quality-attribute considerations. Participants submitted these artifacts electronically after completing the session, using a common submission channel to ensure consistency across both experimental groups.

Using the previously defined performance metrics, we assess the structure, consistency, and adequacy of the selected techniques, with particular attention to how participants balanced completeness and selectivity. The analysis characterizes decision-making behavior under each condition and highlights the effect of structured design support on architectural reasoning.

**Figure 5:**
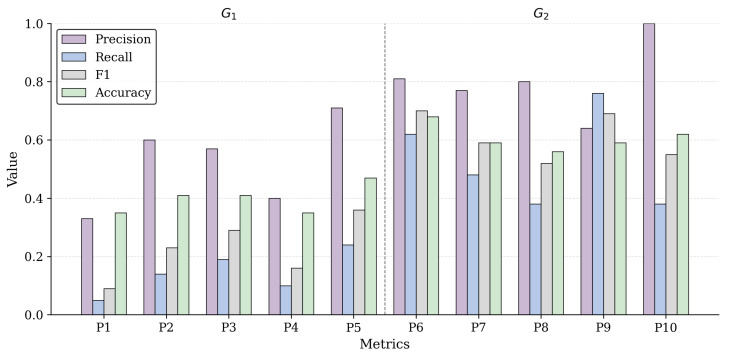
Precision, Recall, F1, and Accuracy values obtained from the experimental validation, organized by metric and study participant.

Table 9 reports the individual outcomes of the experimental task, detailing the TP, TN, FP and FN counts for each participant and the corresponding performance metrics. Figure 5 summarizes the Precision, Recall, F1, and Accuracy values obtained from the experimental validation across study participants.

As seen in Table 9, participants in G_1_ exhibit a consistent pattern: low Recall, low F1, and high false-negative counts. Although Precision is reasonable in some cases, the collective behavior indicates that these practitioners identified only a small portion of the techniques required for an OTA update design. This pattern is entirely expected: without access to the catalog, participants must rely solely on their individual experience, which naturally limits the range of techniques they consider. As a result, their selections show a reduced exploration of the solution space and a tendency to identify only a subset of the techniques relevant to the OTA update system.

On the other hand, participants in G_2_ display a distinctly different behavior. Their Recall is substantially higher, false negatives decrease accordingly, and F1 improves coherently. Accuracy also increases, suggesting that correct selections drive the improvement. These results indicate that enabled participants to conduct a more deliberate examination of the relevant techniques, organizing their decisions around the architectural concerns central to OTA updating rather than ad hoc reasoning.

The contrast between the two conditions shows that the catalog functions as an effective design aid: it broadens the portion of the architectural solution space that participants consider and improves the alignment of their decisions with the GT. The shift is not only quantitative but conceptual, revealing a move from intuition-led choices toward more structured and principled architectural reasoning.

While Table 9 reports the complete set of evaluation outcomes, including TP, TN, FP, and FN counts, Figure 5 provides a complementary visual summary of the derived performance metrics. The column graph highlights the distribution of Precision, Recall, F1, and Accuracy values across individual participants, enabling a direct visual comparison between experimental conditions.

Figure 5 reveals several patterns that are not immediately apparent from the tabular results alone. Most notably, the performance profiles of G_1_ and G_2_ are visually well separated, indicating qualitatively different reasoning behaviors rather than incremental performance differences. Whereas G_1_ exhibits irregular and heterogeneous metric profiles across participants, G_2_ shows more coherent and aligned patterns, particularly for Recall and F1, suggesting increased consistency in architectural decision-making.

The figure also highlights a shift in the relationship between Precision and Recall. In G_1_, moderate Precision coexists with very low Recall, reflecting selective but incomplete reasoning. In contrast, G_2_ achieves higher Recall without a systematic loss of Precision, indicating that the broader coverage is driven by informed selection rather than indiscriminate inclusion. The concurrent improvement and co-movement of Recall, F1, and Accuracy across G_2_ participants further suggests that the catalog reshapes the decision process itself, leading to more systematic and stable architectural reasoning.

#### 6.2.1. Statistical Analysis

Building on the individual-level analysis, we assess whether the observed decision-making patterns persist at the group level. Table 10 reports the aggregated performance metrics for G_1_ and G_2_, including measures of central tendency and dispersion. This aggregation reveals whether per-participant differences represent isolated cases or stable, condition-specific behaviors. By examining the mean, median, and standard deviation of Precision, Recall, F1, and Accuracy, we characterize the dominant reasoning tendencies of each group and assess the catalog’s influence on the overall quality and coherence of the architectural solutions.

The aggregated statistics in Table 10 demonstrate that the two groups follow markedly different decision patterns. G_1_ exhibits consistently low Recall, with both the mean and median at 0.14 and a slight standard deviation of 0.07. This combination indicates a uniform tendency to omit essential techniques, confirming that the behavior observed at the individual level is systematic rather than participant-specific. Precision in G_1_ is moderate, but the pronounced gap between Precision and Recall shows that participants made few selections and rarely identified the techniques required by the GT.

In contrast, G_2_ shows substantially higher Recall (mean 0.52) with increased standard deviation (0.16), a pattern that aligns with the reduction in false negatives observed earlier. This suggests that the catalog enabled a broader and more effective exploration of the design space. At the same time, Precision in G_2_ remains high (mean 0.80) with slightly lower dispersion than G_1_, indicating that improved coverage did not come at the cost of increased false positives. These trends combine to produce a higher and more stable F1 score in G_2_, reflecting a balanced improvement in completeness and correctness.

Accuracy follows the same direction, rising from a mean of 0.40 in G_1_ to 0.61 in G_2_, with similar standard deviation, indicating that the improvement is consistent across participants. Altogether, the statistical profiles indicate that the catalog supports more comprehensive and coherent architectural decision-making, increasing the recovery of relevant techniques while maintaining selection quality and reducing divergence in participant performance.

**Figure 6:**
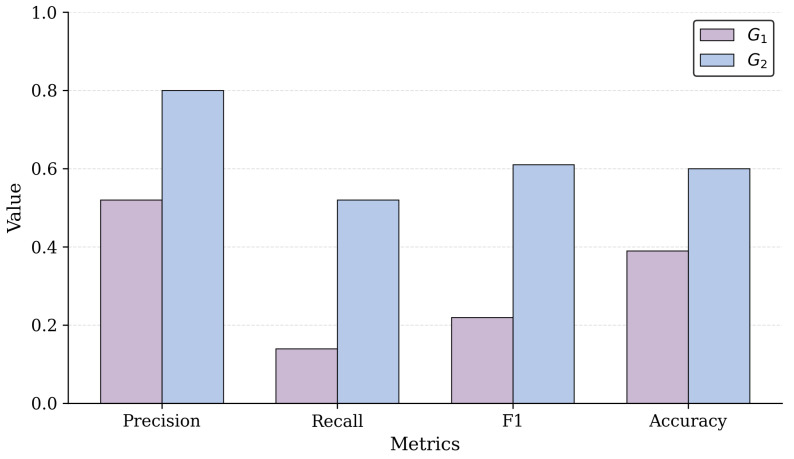
Average values for Precision, Recall, F1, and Accuracy, illustrating the comparative effectiveness across the assessed configurations.

To complement the aggregated statistics reported in Table 10, Figure 6 synthesizes the average values of all evaluation metrics and places them in a directly comparable form. This broader perspective allows us to appreciate how the metrics relate to one another and how consistently they shift across groups. By examining the mean levels of Precision, Recall, F1, and Accuracy side by side, the figure clarifies the overall performance profile produced by the catalog and highlights patterns that are less evident when considering the metrics individually.

Figure 6 shows a clear and consistent performance gap favoring G_2_ across all evaluated metrics. The most pronounced difference appears in Recall, where G_2_ attains values several times higher than G_1_, underscoring the systematic difficulty that unaided participants faced in identifying a sufficient portion of the techniques required for the scenario. The catalog enabled much broader, more complete coverage of the GT, confirming that its impact extends beyond incremental gains.

Precision also increases substantially for G_2_, which indicates that the expanded coverage did not come at the cost of selecting irrelevant techniques. This balance between higher Recall and maintained Precision leads to a pronounced improvement in F1, reflecting more coherent, complete, and structurally aligned architectural solutions. Accuracy shows a similar upward shift, suggesting that the improvements in true positive identification extend to overall classification of relevant and irrelevant techniques, rather than being confined to specific aspects of the task.

A particularly relevant aspect of these aggregated comparisons is that the improvements observed in G_2_ follow a coherent structural pattern rather than appearing as isolated advantages in specific metrics. The parallel increases in Recall, Precision, F1, and Accuracy suggest that the catalog influenced how participants conceptualized the task, guiding them toward a more systematic understanding of which techniques were appropriate for the scenario. This alignment across metrics indicates that the catalog did not merely increase the quantity of techniques selected but also improved the internal consistency of those selections, revealing a qualitative shift in how participants constructed their solutions.

These results outline a coherent advantage for G_2_ and make evident that the improvements observed across metrics are neither marginal nor confined to isolated aspects of the task. Rather, they point to a marked shift in how participants approached identifying relevant techniques.

**Figure 7:**
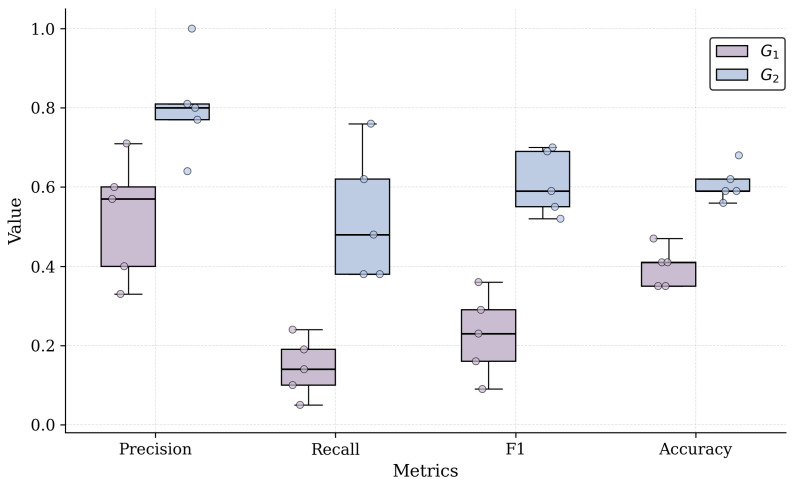
Representation of the distribution values across Precision, Recall, F1, and Accuracy, illustrating the tendency and dispersion observed during the experimental assessment.

Although the previous analyses clarified both individual outcomes and aggregated differences between groups, they do not reveal how evenly these improvements are distributed across participants. Understanding whether the catalog produced a broadly shared shift or whether some participants benefited more than others requires examining the internal distribution of the results. Figure 7 provides this view by displaying the spread and central tendency of each metric, offering insight into the consistency and variability of performance within each group.

As shown in Figure 7, the distributions reveal clear and systematic differences between the two groups. In G_1_, the Recall and F1 values are concentrated near the lower end of the scale, with tight interquartile ranges and no high observations. This pattern indicates that all participants performed at similarly limited levels, consistent with the unaided condition. Without access to the catalog, the range of techniques considered remains narrow, and the resulting solutions reflect uniformly low completeness.

The distributions in G_2_ present a markedly different profile. Recall rises substantially and spans a broader vertical range, indicating that several participants identified a considerably larger subset of relevant techniques once the catalog was available. Precision is higher and more tightly grouped, suggesting a stable ability to identify relevant techniques across participants. The F1 and Accuracy distributions shift upward as well, with boxes positioned clearly above those of G_1_ and only moderate variability, which reflects more balanced and coherent selections in the supported condition.

The boxplots, therefore, provide a distributional view that strengthens the previously observed contrasts. G_1_ remains clustered around low completeness and restricted identification, whereas G_2_ shows consistently higher correctness and broader coverage of the relevant techniques for the scenario. The shape and position of the boxes across metrics indicate that the improvements are systematic rather than isolated, highlighting a meaningful shift in how participants approached the design task when supported by the catalog.

#### 6.2.2. Effect Size Analysis

Beyond examining differences in central tendency and dispersion, it is essential to quantify the magnitude of the observed improvements to determine whether the catalog produces effects of practical significance. Effect sizes characterize the strength of the contrast between G_1_ and G_2_ independently of sample size, making them suitable for small-sample experimental designs [98]. To this end, we computed two non-parametric effect size measures that are standard in empirical software engineering: the Vargha–Delaney A_12_ statistic [99] and Cliff’s δ [100].

For $A_{12}$, we adopted the guidelines of Vargha and Delaney [99], where values near 0.5 indicate negligible dominance and thresholds of 0.56, 0.64, and 0.71 denote small, medium, and large effects, respectively. Cliff’s δ followed the magnitude thresholds introduced by Romano et al. [105], which classify effects as negligible (|δ|<0.147), small (|δ|<0.33), medium (|δ|<0.474), and large (|δ|≥0.474).

These measures capture stochastic dominance between groups and provide interpretable thresholds for judging practical importance. Table 11 summarizes the results for all performance metrics.

The effect size results in Table 11 reveal an exceptionally strong and consistent dominance of the G_2_ across all evaluation metrics. The values of $A_{12}$ and Cliff’s δ fall at the upper end of their respective scales, with every metric classified as a large effect according to established interpretation criteria. This indicates that the improvements observed are not only statistically meaningful but also of substantial practical relevance in the context of architectural decision-making.

The magnitude of these effects follows directly from the structure of the data. In all metrics, every observation in G_2_ exceeds every corresponding observation in G_1_, yielding complete stochastic dominance of the experimental group. Under these conditions, the Vargha–Delaney statistic necessarily attains its upper bound of $A_{12}=1.00$, reflecting that a randomly selected participant from G_2_ will outperform one from G_1_ with probability one. Cliff’s δ mirrors this behavior by reaching δ=1.00, its maximum possible value, indicating that there are no pairwise comparisons in which the control group obtains higher values. These results do not represent anomalies; rather, they constitute the mathematically appropriate outcome when the experimental condition uniformly outperforms the control condition across all observations.

Interpreted within the context of the task, these large effects capture the degree to which the catalog transforms participant performance. For Recall and F1, the dominance values reflect a shift from consistently incomplete solutions in G_1_ to substantially broader and more accurate identification of relevant techniques in G_2_. The large effects observed for Precision and Accuracy further demonstrate that the increased coverage did not introduce instability or noise; instead, the catalog improved both completeness and correctness simultaneously.

When interpreted in relation to the hypotheses defined in Section 6.1.1, the observed effect sizes provide direct empirical evidence regarding the difference between the two experimental conditions. The Vargha–Delaney $A_{12}$ values and Cliff’s δ estimates quantify the magnitude and direction of differences between G_1_ and G_2_ across the evaluated metrics, indicating the extent to which the use of the catalog influences architectural decision-making outcomes.

The consistently non-trivial effect sizes observed for Recall, F1, and overall solution completeness indicate practically meaningful differences between the two groups, supporting the alternative hypothesis ($H_{1}$). In contrast, the null hypothesis ($H_{0}$), which assumes no relevant difference between conditions, is not supported by the observed effect-size magnitudes. Taken together, these results show that the catalog introduces systematic and practically relevant improvements, rather than marginal or incidental variations, in the OTA design task.

## 7. Threats to Validity

This section discusses the main threats to the experiment’s validity and the strategies used to mitigate them, organized according to established categories in empirical software engineering.

### 7.1. Internal Validity

Learning effects may arise because the experimental group received a brief orientation to the catalog’s structure. To minimize this threat, the briefing was strictly procedural and avoided any discussion of OTA techniques or mechanisms relevant to the scenario. All participants received an equivalent clarification on the notion of “architectural technique” to ensure a shared conceptual baseline. Treatment asymmetry, inherent in the design, was controlled by ensuring that the additional information provided to the experimental group did not pre-structure the reasoning needed for the task.

Potential experimenter influence was mitigated through scripted instructions, uniform handling of questions, and the prohibition of technical clarifications during the task. Participants were assigned to groups using stratified randomization based on seniority, reducing systematic differences that could confound the results. All sessions were conducted individually and under supervision to prevent communication, dominance effects, or cross-contamination between participants.

### 7.2. External Validity

The experiment was conducted within a single company, which limits the direct generalizability of the findings to other organizational contexts. This design choice reflects a common constraint in industrial empirical studies, where access to professional practitioners and real-world design settings is inherently limited [98]. To mitigate this threat, the participant pool encompassed diverse areas of specialization and experience levels, reflecting the multidisciplinary composition typically found in industrial IoT engineering teams.

The distribution of technical backgrounds across groups was not controlled beyond seniority stratification and therefore may vary by chance. While this may limit the direct generalization of the results to teams with different profile compositions, it reflects the natural heterogeneity of industrial IoT teams. Moreover, the consistent effect patterns observed across all evaluation metrics suggest that the impact of the catalog is robust to incidental differences in technical background.

In addition, the study focused on a single OTA design scenario, which does not cover the full spectrum of OTA update challenges. This scenario was deliberately constructed to represent a realistic and non-trivial case, incorporating heterogeneous devices, compatibility constraints, operational windows, security requirements, and representative update workflows commonly observed in IoT deployments. While other scenarios may emphasize different quality priorities, the selected case captures core OTA concerns that are broadly applicable across domains.

The individual nature of the task further constrains generalization to collaborative design processes, which are common in industrial practice. This choice was intentional, as it allowed the isolation of individual architectural reasoning and avoided interaction and dominance effects that could confound the comparison between experimental conditions. As a result, the conclusions should be interpreted as evidence of impact under controlled industrial conditions, capturing the direction and practical relevance of the observed effects rather than providing universal predictions for all OTA design contexts.

### 7.3. Construct Validity

The construction of the ground truth (GT) and the assessment of quality attribute impacts relied on expert judgment, which introduces the potential risk of dominance, anchoring, or domain-specific bias. As shown in Table 3, all experts share a strong background in software architecture, reflecting the architectural scope of the study. While this may introduce a software-oriented perspective, the experts’ experience also spans IoT systems, information security, and IT management, providing exposure to deployment, operational, and cross-layer concerns beyond pure software development.

Importantly, the GT and QA evaluation focus on architectural responsibilities and trade-offs rather than low-level implementation or hardware-specific optimizations. From this perspective, a common architectural background supports conceptual consistency in the interpretation of techniques and quality impacts. To mitigate individual bias, the GT definition and QA assessment followed a structured process based on independent evaluations and moderated consensus, with unanimous agreement required for inclusion. As a result, while expert bias cannot be entirely eliminated, its influence is bounded by both the architectural focus of the study and the methodological safeguards applied.

Also, free-form responses from G_1_ required mapping onto catalog techniques, which introduces potential interpretation bias. To address this, each expert performed the mapping independently, followed by a reconciliation process guided by explicit semantic criteria.

The operationalization of performance metrics (Precision, Recall, F1, Accuracy) reflects alignment with the GT rather than absolute architectural optimality. This threat is mitigated by grounding the GT in a transparent, multi-expert methodology and by aligning it with the requirements of the scenario instead of any single architect’s preferences.

### 7.4. Conclusion Validity

With ten participants, the study lacks statistical power for significance testing. To mitigate this limitation, the analysis relies on descriptive statistics and robust nonparametric effect-size estimators such as Vargha–Delaney $A_{12}$ and Cliff’s δ, which remain stable under small sample sizes and non-normal distributions.

To avoid unwarranted generalizations, the conclusions emphasize the strength, coherence, and directionality of the observed effects within the methodological boundaries of the study. The experimental evidence reveals a clear and systematic advantage for participants supported by the catalog, manifested consistently across all evaluation metrics. These results constitute robust and credible evidence in favor of the hypotheses under controlled industrial conditions. While extending these findings to broader contexts requires replication across additional settings, the present experiment provides a rigorous and empirically grounded assessment of the catalog’s impact on architectural decision-making and establishes a strong foundation for future replication studies.

## 8. Discussion

The findings of this study reveal how the DeOTA-IoT catalog reshapes practitioners’ reasoning about OTA update systems when quality attributes and architectural trade-offs are made explicit. The catalog does more than organize existing OTA practices; it exposes the multidimensional nature of OTA design by clarifying how each technique influences the system qualities typically required. This explicit articulation fundamentally alters the decision-making process, particularly in environments where designers must balance security, availability, performance, scalability, and energy constraints under operational and hardware heterogeneity.

The experimental results clearly illustrate this effect. Participants without the catalog relied primarily on personal experience, producing designs that were reasonable but systematically incomplete. Their tendency to omit essential techniques, as reflected in low Recall and high false-negative counts, indicates that unaided reasoning does not naturally recover the full set of responsibilities required for dependable OTA update systems.

In contrast, the catalog enabled participants to navigate the design space more comprehensively. The improvements in Recall and F1, together with more uniform coverage across mechanisms, provide empirical support for $H_{1}$ and demonstrate that the trade-off model is particularly effective in making relevant architectural concerns visible. By externalizing the effects of each technique on ten quality attributes, the catalog encourages designers to reason holistically rather than focusing narrowly on familiar areas such as authentication or secure transport. The reduced variability in G_2_ further indicates that the catalog not only improves decision quality but also stabilizes it by mitigating differences in individual experience within teams.

Importantly, the effect-size analysis demonstrates that these differences are not marginal but practically meaningful, indicating that the catalog substantively influences architectural decision-making rather than merely refining existing intuitions.

The experiment also reveals a deeper insight: the presence of trade-offs does not complicate the design process; it clarifies it. Participants using the catalog were able to understand why specific techniques must be used together, why others compensate for one another’s weaknesses, and why some techniques are necessary even when their benefits are indirect. This suggests that engineers intuitively appreciate quality-driven reasoning when it is explicitly articulated, reinforcing the idea that OTA design benefits from structured guidance rather than ad hoc exploration.

Some techniques still showed patterns of misunderstanding or inconsistent selection, suggesting that further refinement or clearer descriptions could enhance the catalog’s effectiveness. This is unsurprising given the technical diversity of OTA mechanisms and highlights an opportunity for future iterations of the catalog to incorporate examples, constraints, or usage scenarios to support more precise interpretation.

Although the study follows established practices for small-sample industrial experiments, the validation was conducted in a single organization and under a specific OTA scenario. Broader studies in other sectors, such as health, industrial automation, or smart mobility, may reveal how quality priorities shift the relevance of certain techniques or highlight additional trade-offs not captured in this experiment.

The trade-off model deliberately emphasizes quality attributes and does not explicitly model economic or implementation-time costs. This reflects the architectural scope of the study: while quality trade-offs can be reasoned about at a conceptual and system level, cost and effort are inherently context-sensitive and depend on organizational constraints, platform maturity, and deployment scale. Consequently, the catalog is intended to support quality-aware architectural reasoning, with cost considerations treated as a complementary, context-specific dimension that can be incorporated when sufficient situational information is available.

Overall, the results show that the catalog serves as a decision-support instrument that strengthens OTA design by making quality concerns explicit, reducing omissions, and supporting more balanced and consistent reasoning. These insights reinforce the catalog’s role not merely as a reference but as an architectural tool that improves practitioners’ approach to the inherently complex domain of OTA update systems.

## 9. Conclusions and Future Work

This work presents a consolidated architectural foundation for designing OTA update systems in IoT, integrating five years of new evidence into a unified catalog of 34 techniques, six mechanisms, and a trade-off model that makes the quality implications of OTA decisions explicit. Through a rigorous homologation pipeline, expert-driven QA assessment, and an industrial experiment involving professional practitioners, the study demonstrates that the explicit articulation of OTA techniques supports more systematic and higher-quality architectural decisions than unaided experience alone.

Beyond individual contributions, the catalog provides a shared architectural vocabulary that can reduce ambiguity in OTA system design, promote consistency across heterogeneous IoT platforms, and strengthen the rationale for design trade-offs. The experiment confirms that practitioners benefit from this structured guidance even when operating under time constraints and diverse technological backgrounds. These findings offer initial evidence that the catalog can serve as both an analytical instrument and a practical reference for real-world IoT development.

The study also highlights that the value of the catalog extends beyond enumerating techniques: it delineates the interplay between mechanisms, quality attributes, and design responsibilities, offering a foundation for more transparent, traceable, and quality-aware architectural decisions in OTA scenarios. This contribution is particularly relevant in industrial IoT environments, where device heterogeneity, connectivity limitations, and operational constraints require disciplined design practices.

Future research will broaden the empirical validation of the catalog by incorporating larger participant groups, multiple companies, and diverse IoT application domains. Multi-organization and longitudinal studies will strengthen the catalog’s generalizability and inform refinements to its mechanisms and techniques. A complementary direction is the development of an AI-driven decision-support system that operationalizes the catalog. By using its structured metadata and trade-off model, this system would provide context-aware recommendations and automate reasoning about OTA design decisions, enabling practical, large-scale adoption of the catalog in industry.

An important direction for future work is to incorporate economic and implementation-time cost considerations into the OTA techniques catalog. While the present study intentionally focuses on architectural quality attributes to support early-stage design reasoning, economic cost is a critical factor in practical engineering decisions, particularly in resource-constrained IoT environments. Future research may extend the catalog by introducing cost-related dimensions calibrated to specific organizational, platform, or deployment contexts, enabling more comprehensive trade-off analyses that combine quality attributes with economic and effort-based considerations. Such extensions could be supported through empirical studies, industrial case analyses, or parameterized cost models, complementing the quality-driven perspective adopted in this work.

## Figures and Tables

**Table 1:** Digital libraries queried during the literature search.

Name	URL
ACM Digital Library	https://dl.acm.org, 23 November 2025
IEEE Xplore	https://ieeexplore.ieee.org, 23 November 2025
ScienceDirect	https://www.sciencedirect.com, 23 November 2025

**Table 2:** 5-point Likert-style bipolar scale to qualify each QA Impact.

Level	Description	Example
−2	Strong Negative Impact	Blockchain validation severely reduces scalability
−1	Slight Negative Impact	Encryption slightly increases energy consumption
0	Neutral (No Impact)	Metadata format does not affect privacy
1	Slight Positive Impact	Incremental update modestly improves performance
2	Strong Positive Impact	Secure boot ensures firmware reliability

**Table 3:** Experts involved in the assessment of the impact across the QAs in the techniques.

Expert	Experience (Years)	Fields of Expertise
Expert 1	17	Software Architecture, IoT, Information Security
Expert 2	16	Software Architecture, Software Development
Expert 3	17	Software Architecture, IT Management, IoT
Expert 4	13	Software Architecture, Information Security

**Table 4:** Quality Attributes (QAs) in Software Architecture and IoT, built with the QAs listed by [65] and extended with a description for each of them.

ID	QA	Description
QA1	Security	Ability of a system to protect itself from unauthorized access, modifications, or attacks.
QA2	Scalability	Ability of a system to handle increased load or demand without degrading performance.
QA3	Performance	Ability of a system to meet the required speed, responsiveness, and resource usage.
QA4	Availability	Proportion of time that a system is functional and accessible.
QA5	Interoperability	Ability of a system to exchange data and services with heterogeneous devices, platforms, and software components using standardized interfaces, protocols, or formats, requiring minimal adaptation or integration effort.
QA6	Reliability	Ability of a system to consistently perform its required functions under specified conditions.
QA7	Privacy	Ability to safeguard personal and sensitive information collected by devices, ensuring it is only used for its intended purpose.
QA8	Energy Management	Ability to use energy efficiently across devices, ensuring optimal performance while minimizing power consumption.
QA9	Flexibility	Ability of a system to adapt to future requirements or changes in environment with minimal disruption.
QA10	Evolvability	Ability of a system to accommodate functional, technological, or operational changes over time with limited architectural restructuring, low implementation effort, and controlled impact on existing system behavior.

**Table 5:** Techniques and their corresponding QAs’ impact, organized by mechanisms and specified with each technique ID.

Mechanism	Technique	QA1	QA2	QA3	QA4	QA5	QA6	QA7	QA8	QA9	QA10
M1	T1.1	+2	+1	0	+1	+1	+2	0	−1	−1	0
	T1.2	+2	+1	−1	+1	+1	+1	+2	−1	+1	0
	T1.3	+2	−1	0	+1	−1	+2	+1	−1	−1	+1
	T1.4	+2	+1	−1	+1	+2	+2	+2	−1	+1	+1
	T1.5	+2	−2	−1	+1	−1	+1	−1	−2	+1	+1
	T1.6	+2	+1	−1	+1	+1	+2	0	−1	+1	+1
	T1.7	+2	+1	0	+1	+1	+2	+1	−1	+1	+1
	T1.8	+2	+1	0	+2	−1	+2	+1	−1	−1	+1
M2	T2.1	+1	+2	+1	+1	+2	+1	0	0	+1	+1
	T2.2	+2	+1	0	+1	+1	+1	+1	0	+1	+1
	T2.3	+1	+1	0	+1	+1	+2	0	0	+1	+1
	T2.4	+1	+2	+1	+1	+1	+1	0	+2	+2	+1
	T2.5	+1	0	0	+1	0	+1	+2	0	+1	+1
M3	T3.1	+1	+2	+1	+1	+2	+1	0	+1	+2	+1
	T3.2	+2	+1	+1	+1	+1	+2	0	0	+1	+2
	T3.3	+1	+1	+1	+2	0	+2	0	−1	0	+1
	T3.4	+1	+1	+1	+1	+1	+2	0	−1	+1	+1
	T3.5	0	+1	+2	+1	0	+1	0	+2	+1	+1
	T3.6	+2	+1	+1	+1	+1	+1	+1	−1	+1	+1
M4	T4.1	+1	+1	+1	+2	0	+2	0	−1	+1	+1
	T4.2	+1	+1	0	+2	0	+2	0	−1	+1	+1
	T4.3	+2	+1	0	+1	0	+1	+1	0	+1	+1
	T4.4	0	+1	+1	+1	0	+1	0	+2	+1	+1
	T4.5	+1	+1	0	+2	0	+2	0	−1	+1	+1
M5	T5.1	0	+1	+1	+2	0	+2	0	+2	+1	+1
	T5.2	+1	+1	0	+1	0	+1	0	0	+1	+1
	T5.3	0	+1	+1	+2	0	+1	0	+1	+2	+1
	T5.4	+2	+1	0	+1	0	+1	+1	0	+1	+1
	T5.5	+2	+1	+1	+1	0	+2	0	0	+1	+2
M6	T6.1	+1	+1	+1	0	+2	+1	0	0	+1	+1
	T6.2	+1	+1	+1	+1	0	+1	0	+1	+2	+2
	T6.3	+1	+1	+1	+1	+1	+1	0	0	+1	+2
	T6.4	0	+1	+2	+1	0	+1	0	+2	0	+1
	T6.5	+2	+1	−1	+1	+1	+1	0	+1	+1	+1

Legend: QA1: Security; QA2: Scalability; QA3: Performance; QA4: Availability; QA5: Interoperability; QA6: Reliability; QA7: Privacy; QA8: Energy Management; QA9: Flexibility; QA10: Evolvability; M1: Mechanism for secure and safe updates; M2: Mechanism for updates management; M3: Mechanism for code dissemination; propagation, and installation, M4: Mechanism for system recovery; M5: Mechanism for updates scheduling; M6: Mechanism for elaboration and packaging.

**Table 6:** Participants, experience, and technical background.

Group ID	Participant ID	Experience (Years)	Technical Domain
G_1_	P1	13	Web and Mobile Development
	P2	2	Software Development
	P3	1	Software Development
	P4	1	Firmware Development
	P5	5	Firmware Development
G_2_	P6	14	Hardware
	P7	14	Software, Telematics and Cloud
	P8	1	Hardware
	P9	2	FPGA, DSP, and Embedded Systems
	P10	2	FPGA

Legend: FPGA: Field-Programmable Gate Array; DSP: Digital Signal Processor.

**Table 7:** Structure and timing of the experimental session.

Activity	Description	Duration
1. Introduction & Training	Presentation of the experiment’s objectives, group allocation, and a short training on the concept of architectural techniques to ensure a shared understanding of terminology.	15 min
2. Materials Delivery	Distribution of the complete materials package: OTA design scenario, detailed written instructions, and standardized response templates to both groups. The experimental group additionally received the catalog.	10 min
3. Catalog Briefing (G_2_ only)	Orientation on the catalog’s structure, contents, and examples of technique selection for the experimental group.	10 min
4. Design Activity	Independent design of the OTA subsystem. The experimental group (G_2_) applied the catalog during the task, while the control group (G_1_) relied solely on professional experience.	60 min
5. Debriefing & Submission	Submission of the selected catalog techniques for the OTA scenario, followed by a brief feedback session.	15 min

**Table 8:** Metrics utilized in analyzing the experimental validation of the techniques catalog.

Metric	Description	Formula
Precision	The ratio of true-positive selections to the total number of techniques chosen by a participant, indicating the correctness of their selections.	(7) $\mathrm{Precision}=\frac{TP}{TP+FP}$
Recall	The proportion of techniques present in the GT that were correctly selected.	(8) $\mathrm{Recall}=\frac{TP}{TP+FN}$
F1	A harmonic-mean aggregation of Precision and Recall that captures their joint performance in a single, balanced indicator.	(9) $F1=2\times \frac{\mathrm{Precision}\times \mathrm{Recall}}{\mathrm{Precision}+\mathrm{Recall}}$
Accuracy	The proportion of all evaluated techniques for which the participant made the correct decision, combining both true positives and true negatives.	(10) $\mathrm{Accuracy}=\frac{TP+TN}{TP+TN+FP+FN}$

Legend: TP: True Positives; TN: True Negatives; FP: False Positives; FN: False Negatives.

**Table 9:** Results of the experimental validation, organized by metrics and study participants.

Metric	$G_{1}$					$G_{2}$				
	P1	P2	P3	P4	P5	P6	P7	P8	P9	P10
TP	1	3	4	2	5	13	10	8	16	8
TN	11	11	10	10	11	10	10	11	4	13
FP	2	2	3	3	2	3	3	2	9	0
FN	20	18	17	19	16	8	11	13	5	13
Precision	0.33	0.60	0.57	0.40	0.71	0.81	0.77	0.80	0.64	1.00
Recall	0.05	0.14	0.19	0.10	0.24	0.62	0.48	0.38	0.76	0.38
F1	0.09	0.23	0.29	0.16	0.36	0.70	0.59	0.52	0.69	0.55
Accuracy	0.35	0.41	0.41	0.35	0.47	0.68	0.59	0.56	0.59	0.62

**Table 10:** Descriptive comparison of evaluation metrics between G_1_ and G_2_.

Metric	Statistic	G_1_	G_2_
Precision	Mean	0.52	0.80
	Median	0.57	0.80
	Std. Dev	0.15	0.13
Recall	Mean	0.14	0.52
	Median	0.14	0.48
	Std. Dev	0.07	0.16
F1	Mean	0.23	0.61
	Median	0.23	0.59
	Std. Dev	0.11	0.08
Accuracy	Mean	0.40	0.61
	Median	0.41	0.59
	Std. Dev	0.05	0.05

**Table 11:** Effect size results for the comparison between G_1_ and G_2_ using Vargha–Delaney A_12_ and Cliff’s δ, including separate magnitude interpretations.

Metric	A_12_	Cliff’s δ	Effect Size (A_12_)	Effect Size (δ)
Precision	0.96	0.92	Large	Large
Recall	1.00	1.00	Large	Large
F1	1.00	1.00	Large	Large
Accuracy	1.00	1.00	Large	Large

## Data Availability

The original data presented in the study are openly available in Zenodo at https://doi.org/10.5281/zenodo.17404292.
